# Endothelial Activation and Stress Index (EASIX) Predicts In-Hospital Mortality in Acute Decompensated Heart Failure with Reduced Ejection Fraction

**DOI:** 10.3390/diagnostics16010152

**Published:** 2026-01-02

**Authors:** Bülent Özlek, Veysel Ozan Tanık, Alperen Taş, Süleyman Barutçu, Buse Çuvalcıoğlu, Çağatay Tunca, Kürşat Akbuğa, Yusuf Bozkurt Şahin, Murat Akdoğan

**Affiliations:** 1 Department of Cardiology, School of Medicine, Mulla Sıtkı Koçman University, Muğla 48000, Turkey; 2 Department of Cardiology, Ankara Etlik City Hospital, Ankara 06170, Turkey; 3 Department of Cardiology, Kırşehir Training and Research Hospital, Kırşehir 40100, Turkey

**Keywords:** acute decompensated heart failure, endothelial dysfunction, EASIX score, in-hospital mortality, risk stratification

## Abstract

**Background:** Early risk stratification in acute decompensated heart failure with reduced ejection fraction (ADHF-rEF) remains challenging. The Endothelial Activation and Stress Index (EASIX)—a composite of lactate dehydrogenase, creatinine, and platelet count—reflects endothelial dysfunction, a pathophysiological contributor to early deterioration in ADHF-rEF. This study evaluated the prognostic utility of admission-based EASIX for in-hospital mortality. **Methods:** In this retrospective single-center cohort, 850 consecutive patients hospitalized with ADHF-rEF between January 2022 and June 2025 were analyzed. EASIX was calculated from first-day laboratory values. Logistic regression, ROC analysis, restricted cubic splines, and Kaplan–Meier survival methods were used to assess the association between EASIX and in-hospital mortality, and to evaluate its incremental value beyond established clinical and laboratory predictors. **Results**: In-hospital mortality was 12.4%. Higher EASIX values were significantly associated with mortality in both univariable and multivariable models (adjusted OR 1.273; *p* < 0.001). EASIX demonstrated moderate discriminative performance among evaluated biomarkers (AUC 0.751) and showed a clear dose–response risk gradient, with mortality rising from 1.4% in the lowest tertile to 26.2% in the highest. Incorporating EASIX into clinical and laboratory prediction models yielded substantial continuous net reclassification improvement (0.59 and 0.38, respectively). Survival curves diverged early and remained distinctly separated across EASIX strata. **Conclusions:** Admission EASIX is an independent predictor of in-hospital mortality in ADHF-rEF and provides complementary prognostic information beyond conventional models. This is the first study to demonstrate the prognostic value of EASIX in the ADHF-rEF setting, supporting its potential utility as an accessible endothelial stress biomarker for early risk stratification.

## 1. Introduction

Acute decompensated heart failure due to reduced left ventricular ejection fraction (ADHF-rEF) continues to impose high rates of hospitalization and significant early mortality despite modern advances in guideline-directed therapies [1]. Reported in-hospital mortality for ADHF-rEF typically ranges from 2% to 20%, underscoring a persistent unmet need for rapid and reliable admission-based risk stratification, particularly in resource-constrained and high-wound clinical settings [2]. Current prognostic frameworks in ADHF-rEF integrate clinical features, comorbidities, congestion status, natriuretic peptides, renal indices, inflammatory biomarkers, electrolytes, and hematologic measures [3,4]. Established clinical risk factors—including advanced age, diabetes mellitus (DM), chronic kidney disease (CKD), and coronary artery disease (CAD)—remain dominant predictors of adverse outcomes [5]. Nonetheless, existing bedside tools incompletely capture acute systemic stress and multiorgan vulnerability at the time of decompensation.

Endothelial dysfunction represents a mechanistically plausible driver of early deterioration in ADHF-rEF [6]. Hemodynamic overload, oxidative injury, inflammation, and microcirculatory imbalance converge on the endothelium, promoting tissue hypoxia, thrombogenicity, and compromised organ perfusion [7]. Although direct assays for endothelial injury have demonstrated prognostic relevance [8], their cost, complexity, and turnaround time limit real-world application. Therefore, composite, laboratory-based surrogates that approximate systemic endothelial stress may bridge a clinically meaningful gap. The Endothelial Activation and Stress Index (EASIX), derived from lactate dehydrogenase (LDH), platelet count, and serum creatinine, was initially proposed as a simple, accessible marker associated with endothelial activation [9]. Beyond its transplantation origins [9], EASIX has demonstrated external validity across diverse cardiovascular and critical systemic conditions, including chronic heart failure (HF) [10] and coronary syndromes [11], in which elevated values signal greater systemic injury and increased mortality risk. Its components reflect key axes of ADHF-rEF biology: renal dysfunction, cellular turnover and hypoxic injury, and hematologic stress or immunothrombotic imbalance [12]. However, evidence for EASIX in ADHF-rEF remains sparse. The ability of EASIX to independently discriminate in-hospital mortality and to add incremental value over conventional markers has not been thoroughly defined. Therefore, the present study is specifically focused on patients hospitalized with ADHF-rEF, rather than chronic stable HF populations.

We hypothesized that EASIX measured at hospital admission would serve as an independent predictor of in-hospital mortality in ADHF-rEF and enhance the prognostic performance of multivariable clinical and laboratory-based models. We assessed this hypothesis in a consecutive real-world cohort of hospitalized patients with ADHF-rEF, focusing on EASIX’s association with mortality and its additive utility to established prognostic axes.

## 2. Methods

### 2.1. Study Design and Setting

This retrospective, single-center observational analysis was performed at Ankara Etlik City Hospital, a high-volume tertiary referral institution. All patients were screened through the hospital’s electronic health record (EHR) database, and consecutive admissions with a primary discharge diagnosis of ADHF-rEF between January 2022 and June 2025 were evaluated. HF diagnosis and phenotypic classification were based on the 2021 European Society of Cardiology Guidelines for the diagnosis and treatment of acute and chronic HF [13]. ADHF-rEF was defined as a rapid or gradual onset of symptoms and/or signs resulting in hospitalization and requiring urgent medical therapy, typically due to systemic or pulmonary congestion, with or without hypoperfusion. HF with reduced ejection fraction (HFrEF) was defined as left ventricular ejection fraction (LVEF) ≤40% in the presence of clinical HF syndrome and documented structural or functional cardiac abnormality. Accordingly, the study population included both patients with newly diagnosed HFrEF and those with pre-existing chronic HFrEF who were hospitalized due to acute decompensation. During hospitalization, all patients were treated in accordance with contemporary ESC guideline-recommended therapy for ADHF-rEF [13], with treatment strategies individualized based on clinical presentation and hemodynamic status.

### 2.2. Eligibility and Exclusion Criteria

**Figure 1:**
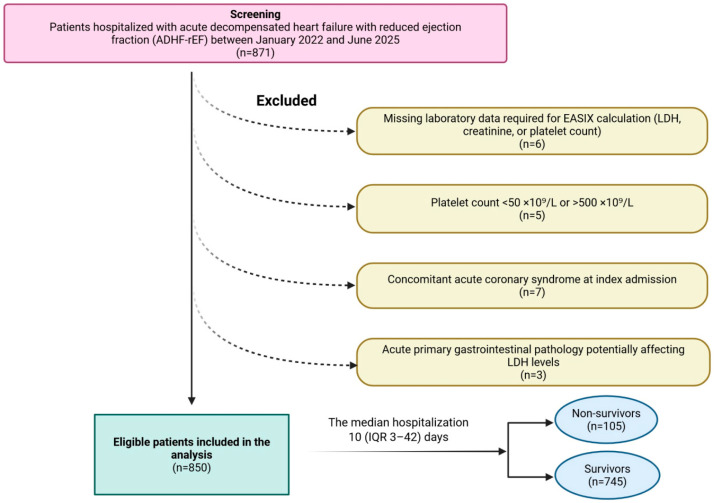
Flow chart of patient selection and study population. Abbreviations: EASIX, endothelial activation and stress index; IQR, interquartile range; LDH, lactate dehydrogenase.

The study included adult patients admitted with a confirmed diagnosis of ADHF-rEF, provided that all laboratory parameters required for the EASIX calculation were measurable within the first 24 h of hospitalization. Patients meeting any of the following conditions were excluded: absence of LDH, platelet or serum creatinine values in the EHR system (*n* = 6); platelet counts below 50 × 10^9^/L or above 500 × 10^9^/L to minimize confounding by potential occult hematologic disease (*n* = 5); concurrent acute coronary syndrome at index admission (*n* = 7); or admission with acute primary gastrointestinal pathology that could independently alter LDH levels (*n* = 3). A total of 21 patients were removed prior to analysis, leaving 850 eligible patients for the final cohort (Figure 1).

### 2.3. Ethical Considerations

Approval for the study was obtained from the Ankara Etlik City Hospital Local Clinical Research Ethics Committee (Approval number and date: 3 September 2025—AEŞH-BADEK1-2025-438). Ethics committee approval was obtained prior to data extraction and analysis; given the study’s retrospective, non-interventional nature, the requirement for written informed consent was waived. All procedures and data handling were compliant with the ethical principles of the Declaration of Helsinki and institutional data protection policies. Patient information was anonymized prior to extraction, and only routinely recorded clinical data were analyzed.

### 2.4. Data Collection and Clinical Variables

Demographic characteristics, past medical history, chronic cardiovascular conditions, pre-admission medication use, and laboratory findings were retrieved through structured EHR queries and manually verified using physician-documented discharge summaries, hospital epicrisis records, and diagnostic coding tables. The presence of comorbid conditions was determined by ICD-10 diagnostic codes assigned at discharge and confirmed by supporting clinical documentation in medical records, without requiring extensive phenotypic subclassification. Laboratory measurements comprised complete blood count, serum electrolytes, and renal and hepatic biochemical panels, all routinely obtained within the first 24 h after admission. Inflammatory status at presentation was evaluated using C-reactive protein (CRP), which had been included in the hospital’s standard laboratory order set during the study period.

### 2.5. Echocardiographic Assessment

Transthoracic echocardiography was conducted at hospital admission by licensed cardiologists or senior cardiology fellows working under direct consultant supervision. Imaging protocols were aligned with American Society of Echocardiography recommendations for chamber quantification and systolic function assessment [14]. A Hitachi ultrasound cardiovascular platform (Arietta 65, Twinsburg, OH, USA) equipped with a 2.5–3.5 MHz phased-array transducer was used for all examinations. LVEF was measured using the biplane Simpson’s method from apical 4-chamber and 2-chamber windows, ensuring methodological consistency across patients. All echocardiographic measures analyzed were derived from the first clinically documented study of hospitalization.

### 2.6. EASIX Calculation

EASIX was computed from admission laboratory values using the formula [10]:

EASIX = LDH (U/L) × Creatinine (mg/dL) / Platelet count (10^9^/L)

This index was not used in clinical decision-making at the time of admission and was calculated offline for research analysis after data extraction.

### 2.7. Outcome and Follow-Up

The principal endpoint was in-hospital all-cause mortality, as captured from medical records. Patients were followed from admission until mortality or discharge, whichever occurred first. To explore differences in early clinical risk profile, survivors and non-survivors were compared with respect to admission vital signs, laboratory derangements, and echocardiographic-derived systolic performance. Comparisons aimed to reflect the biological and clinical divergence associated with early mortality in ADHF-rEF.

### 2.8. Statistical Analyses

Continuous variables were assessed for normality using the Shapiro–Wilk test and were presented as mean ± standard deviation or median with interquartile range, as appropriate. Categorical variables were summarized as counts and percentages. Comparisons between two groups were performed using the independent-samples *t*-test or the Mann–Whitney U test for continuous variables, and the χ^2^ test for categorical variables. Comparisons across EASIX tertiles (three groups) were performed using one-way ANOVA for normally distributed continuous variables or the Kruskal–Wallis test for non-normally distributed variables. Categorical variables were compared using the χ^2^ test or Fisher’s exact test as appropriate. Univariable logistic regression was used to examine the association between each clinical and laboratory variable and in-hospital mortality. Variables demonstrating clinical relevance or a *p*-value less than 0.10 in univariable analysis were considered for multivariable modeling. Platelet count, serum creatinine, and LDH were excluded from the multivariable model to avoid multicollinearity, as these parameters constitute the components of the EASIX formula. In addition, admission blood glucose was not entered simultaneously with DM because of their close clinical and metabolic interrelationship. Multicollinearity among variables in the final multivariable model was formally assessed using variance inflation factors (VIFs) and tolerance statistics. All variables demonstrated acceptable collinearity indices (VIF < 2.5; tolerance > 0.4), indicating no relevant multicollinearity. Multivariable logistic regression, including age, DM, CAD, CKD, serum sodium, hemoglobin, CRP, N-terminal pro–B-type natriuretic peptide (NT-proBNP) (scaled per 500-unit increment), and the EASIX score, was performed to identify independent predictors of in-hospital mortality. Results were presented as odds ratios (ORs) with corresponding 95% confidence intervals (CIs). Receiver operating characteristic (ROC) analyses were conducted to evaluate the discriminative performance of the EASIX score and other predictors. Areas under the curve (AUC) were compared using De Long’s test. Optimal cut-off values for the EASIX score were obtained using Youden’s index. Incremental prognostic contribution of the EASIX score beyond clinical and laboratory models was assessed using continuous net reclassification improvement (NRI). Potential nonlinear associations between the EASIX score and mortality were explored using restricted cubic spline (RCS) logistic regression with three knots. Model fit and nonlinearity were evaluated using likelihood ratio testing. The prespecified EASIX threshold derived from ROC analysis was indicated on spline curves for interpretability. In-hospital survival was evaluated using Kaplan–Meier methods. Patients were stratified according to the optimal cut-off (EASIX score < 1.246 vs. ≥1.246) and by EASIX score tertiles (≤0.677, 0.678–1.466, ≥1.466). Survival differences were tested using the log-rank test. Hazard ratios (HRs) and 95% CIs comparing high- vs. low-EASIX groups were estimated using Cox proportional hazards models. Cox proportional hazards models were used to illustrate in-hospital survival differences and to account for variation in length of hospital stay. These analyses were intended to be descriptive and supportive rather than for primary risk estimation. The proportional hazards assumption was formally assessed using Schoenfeld residuals and was not violated for the EASIX-based models (all *p* > 0.05). For tertile analyses, overall log-rank statistics and pairwise comparisons were computed. All Kaplan–Meier curves were displayed with 95% confidence bands. All analyses were conducted using SPSS (version 26.0, IBM Corp., Armonk, NY, USA) and Python (version 3.14). A two-sided *p*-value less than 0.05 was accepted as statistically significant.

## 3. Results

Among screened admissions for ADHF-rEF, 850 patients met all inclusion criteria and were included in the final analysis. Overall, in-hospital all-cause mortality was 12.4% (*n* = 105). The study population was predominantly male (71.5%), reflecting the sex distribution typical of a high-acuity HFrEF cohort. The median hospitalization duration for the full sample was 10 days (IQR 3–42).

The clinical and laboratory characteristics of patients who survived and those who died during hospitalization are presented and compared in Table 1. The mean age of hospitalized survivors was 63.7 ± 13.2 years. Patients who died in-hospital were older, with a mean age of 71.8 ± 11.2 years (*p* < 0.001). Sex distribution was similar between groups, with males comprising 74.3% of non-survivors and 71.1% of survivors. Smoking prevalence did not differ significantly between groups, and initial vital signs such as systolic blood pressure and heart rate were also comparable. A significantly higher proportion of non-survivors had DM (53.3 vs. 35.6%, *p* < 0.001), CAD (61.9 vs. 44.4%, *p* = 0.001), and CKD (45.7 vs. 30.1%, *p* = 0.001). The prevalence of other comorbidities, including hypertension, atrial fibrillation, peripheral artery disease, chronic obstructive pulmonary disease (COPD), and the presence of cardiac devices, did not differ significantly between the groups. Baseline use of major HF medications—including β-blockers, angiotensin converting enzyme inhibitors or angiotensin II receptor blockers, mineralocorticoid receptor antagonists, antiplatelets, anticoagulants, and statins—was similar between survivors and non-survivors, with no statistically significant differences observed (all *p* > 0.05). Laboratory evaluation at admission revealed notable disparities in several parameters. Non-survivors had significantly higher median NT-proBNP levels (2016 vs. 1070 pg/mL, *p* < 0.001), serum creatinine (1.6 vs. 1.1 mg/dL, *p* < 0.001), LDH (221.6 ± 25.1 vs. 207.1 ± 40.4 U/L, *p* < 0.001), CRP (23 vs. 15 mg/L, *p* < 0.001), and blood glucose. Conversely, hemoglobin levels were significantly lower among non-survivors (11.8 ± 1.6 vs. 13.1 ± 2.4 g/dL, *p* < 0.001), as were serum sodium levels (136.7 ± 4.3 vs. 138.0 ± 4.3 mmol/L, *p* = 0.004). Platelet counts were slightly lower in non-survivors, though the difference was not statistically significant (*p* = 0.080). Median EASIX score was also significantly elevated in non-survivors (1.49 vs. 0.94, *p* < 0.001). Lastly, hospitalization was longer in non-survivors than in survivors (median 13 vs. 9 days, *p* = 0.001).

### 3.1. Predictors of In-Hospital Mortality

As presented in Table 2, both univariable and multivariable logistic regression analyses identified several predictors of in-hospital mortality among patients with ADHF-rEF. In univariable analysis, advanced age was significantly associated with mortality risk, and this association persisted in multivariable modeling (OR: 1.045; 95% CI: 1.025–1.065; *p* < 0.001). DM conferred a two-fold increased risk of mortality (univariable OR: 2.070; *p* < 0.001; multivariable OR: 2.115; *p* = 0.001). Similarly, CKD remained a significant independent predictor in the adjusted model (OR: 1.652; 95% CI: 1.039–2.628; *p* = 0.034). Among laboratory variables, higher NT-proBNP levels (per 500 pg/mL) were consistently predictive of mortality (multivariable OR: 1.020; *p* = 0.021). Lower serum sodium was inversely associated with mortality risk (multivariable OR: 0.945; *p* = 0.025). Hemoglobin levels were inversely related to in-hospital mortality (multivariable OR: 0.832; *p* < 0.001). Inflammatory burden, as reflected by CRP levels, was also associated with increased mortality risk (multivariable OR: 1.005; 95% CI: 1.002–1.008; *p* < 0.001). In particular, the EASIX emerged as an independent predictor of in-hospital mortality. An increase in EASIX score was associated with a 27.3% increase in the odds of in-hospital mortality (multivariable OR: 1.273; 95% CI: 1.123–1.442; *p* < 0.001).

**Figure 2:**
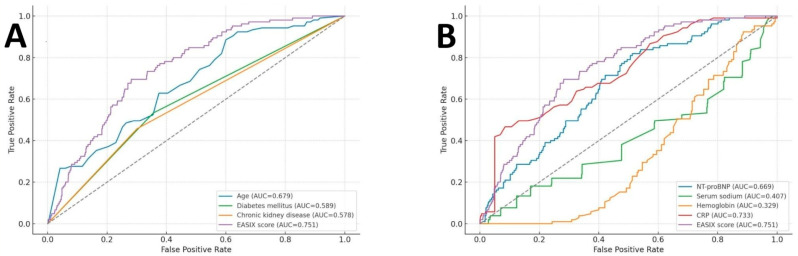
(**A**) Receiver operating characteristic curves for clinical parameters including age, diabetes mellitus, chronic kidney disease, and the EASIX score in predicting in-hospital mortality. The EASIX score demonstrated moderate discriminative performance (AUC = 0.751); (**B**) ROC curves for laboratory parameters, including N-terminal pro–B-type natriuretic peptide (NT-proBNP), serum sodium, hemoglobin, C-reactive protein (CRP), and the EASIX score, in predicting in-hospital mortality. Among all laboratory markers, the EASIX score demonstrated the highest discriminative ability (AUC = 0.751). Abbreviations: AUC, the area under the curve; EASIX, endothelial activation and stress index.

Figure 2 illustrates the ROC curves evaluating the predictive performance of EASIX and other variables for in-hospital mortality. Among all evaluated parameters, the EASIX score demonstrated moderate discriminative capacity, with an AUC of 0.751 (95% CI: 0.707–0.795; *p* < 0.001). Based on Youden’s index, an optimal EASIX cut-off value of 1.246 was identified, corresponding to a sensitivity of 69.5%, specificity of 69%, negative predictive value (NPV) of 94.1%, and positive predictive value (PPV) of 24.0%. In Figure 2A, EASIX outperformed conventional clinical risk factors, including age, DM, and CKD. In Figure 2B, the EASIX score also surpassed key laboratory indicators, including NT-proBNP, serum sodium, hemoglobin, and CRP.

**Figure 3:**
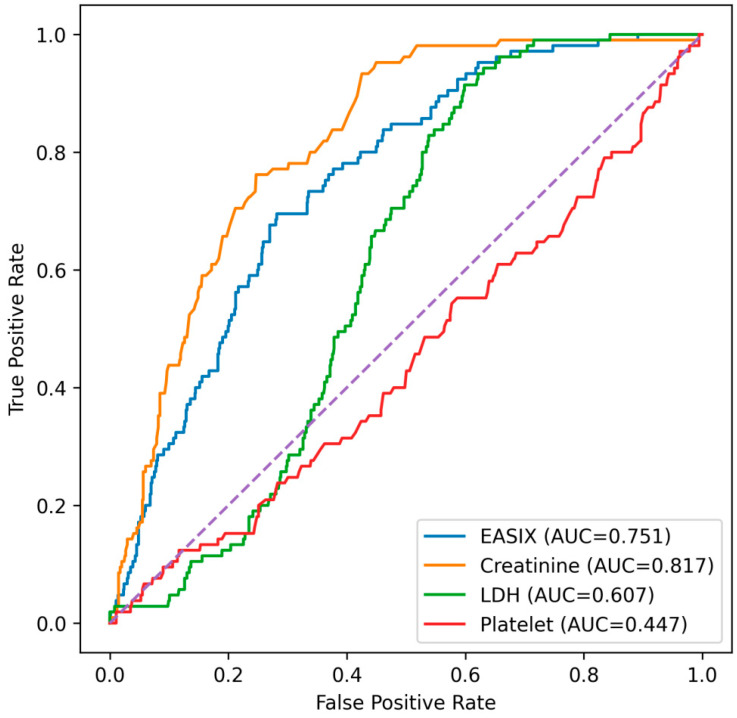
Receiver operating characteristic curves comparing the predictive performance of the EASIX score, serum creatinine, LDH, and platelet count for in-hospital mortality. Serum creatinine demonstrated the highest discriminative ability, followed by the EASIX score. LDH showed moderate performance, whereas platelet count alone had no significant predictive value. Abbreviations: AUC, the area under the curve; EASIX, endothelial activation and stress index; LDH, lactate dehydrogenase.

Given that the EASIX score is composed of serum creatinine, LDH, and platelet count, additional ROC analyses were performed to compare its discriminative performance with that of its individual components (Figure 3). Among these parameters, serum creatinine demonstrated the highest discriminative ability, with an AUC of 0.815. The EASIX score also showed good predictive performance, yielding an AUC of 0.751. In contrast, LDH exhibited modest discrimination (AUC = 0.606), while platelet count alone showed no meaningful predictive value (AUC = 0.446). Serum creatinine showed a significantly higher AUC than the EASIX score (DeLong *p* < 0.001). In contrast, the EASIX score demonstrated significantly higher discriminative performance than LDH (DeLong *p* < 0.001) and platelet count (DeLong *p* < 0.001).

### 3.2. Incremental Value of EASIX Beyond Conventional Predictors

**Figure 4:**
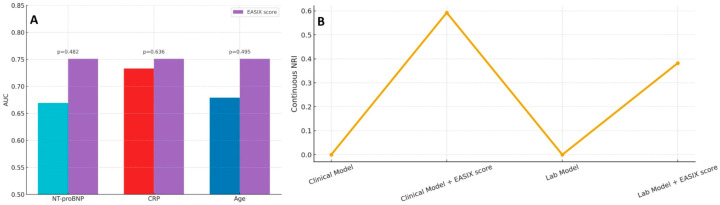
(**A**) Comparison of the discriminative performance of the EASIX score versus established predictors (NT-proBNP, CRP, and age). Although the EASIX score showed numerically higher AUC values, differences were not statistically significant according to De Long’s test; (**B**) Continuous net reclassification improvement (NRI) showing the incremental prognostic value of adding the EASIX score to the clinical model and to the laboratory model. Incorporation of the EASIX score resulted in marked reclassification gains for both model types. Abbreviations: AUC, the area under the curve; EASIX, endothelial activation and stress index; CRP, C-reactive protein; NT-proBNP, N-terminal pro–B-type natriuretic peptide.

As shown in Figure 4A, the EASIX score yielded a higher AUC than individual variables such as NT-proBNP, CRP, and age; however, these differences did not reach statistical significance based on DeLong’s test. Nevertheless, when incorporated into multivariable prediction frameworks, EASIX provided meaningful incremental prognostic value. As shown in Figure 4B, adding EASIX to a baseline clinical model substantially improved risk discrimination, as reflected by a continuous NRI of approximately 0.59. Similarly, integrating EASIX into a laboratory-based model yielded a continuous NRI of 0.38, supporting its complementary utility in refining risk stratification for in-hospital mortality.

### 3.3. Dose-Dependent Association Between EASIX and Mortality Risk

**Figure 5:**
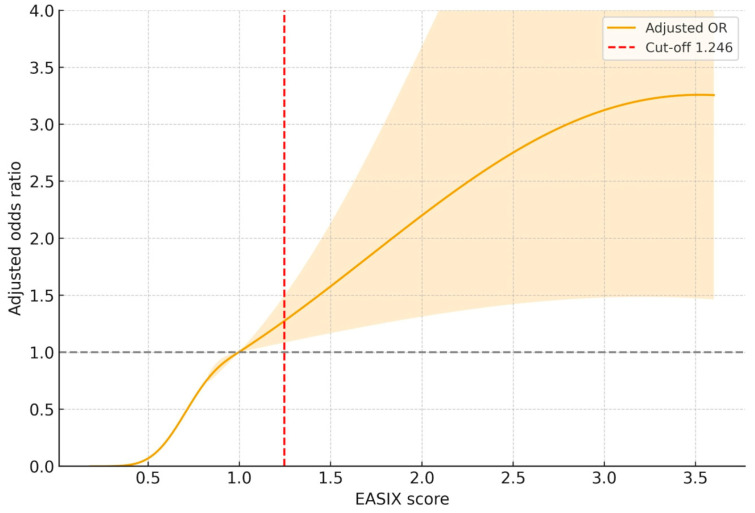
Restricted cubic spline analysis depicting the adjusted association between EASIX score and in-hospital mortality. The spline model was adjusted for age, diabetes mellitus, chronic kidney disease, NT-proBNP, serum sodium, hemoglobin, and C-reactive protein. The median EASIX score (~1.0) was used as the reference (OR = 1). The curve demonstrates a significant non-linear relationship between EASIX and mortality risk (*p* for non-linearity < 0.001). A vertical dashed line marks the optimal cut-off (EASIX = 1.246). Shaded areas represent 95% confidence intervals. Abbreviations: EASIX, endothelial activation and stress index.

As depicted in Figure 5, RCS modeling revealed a non-linear, progressively increasing association between the EASIX score and the adjusted odds of in-hospital mortality. The risk began to rise notably above an EASIX value of approximately 1.0, and crossed the reference threshold (OR = 1.0) around the cut-off point of 1.246, consistent with ROC-derived results. Beyond this cut-off, the curve showed a dose-dependent escalation in adjusted odds ratio, peaking around an EASIX value of 3.5. The 95% CI remained above unity across this higher range.

### 3.4. Clinical and Laboratory Characteristics Across EASIX Tertiles

As presented in Table 3, patients were stratified into tertiles based on their EASIX scores. A progressive increase in age was observed across tertiles, with patients in the highest tertile significantly older than those in the lowest tertile (*p* < 0.001). Male predominance also increased significantly across tertiles (*p* < 0.001). Regarding comorbid conditions, the prevalence of CAD and CKD rose significantly with increasing EASIX scores. Similarly, the proportion of patients with COPD was highest in the upper tertile compared to the lower tertile. Although DM and hypertension were more frequent in higher EASIX groups, these differences did not reach statistical significance. In terms of pharmacological therapy, β-blocker and anticoagulant use were significantly higher among patients in the upper tertiles. Laboratory parameters revealed a graded decline in serum albumin, serum sodium, hemoglobin, and lymphocyte counts with increasing EASIX scores. Additionally, blood glucose, serum creatinine, CRP levels, and NT-proBNP concentrations were significantly elevated in higher EASIX groups. Importantly, in-hospital mortality rates showed a marked stepwise increase from the lowest to the highest tertile (1.4 vs. 10.8 vs. 26.2%, *p* < 0.001), highlighting a graded association between EASIX tertiles and in-hospital mortality. However, hospitalization length did not differ significantly across tertiles.

### 3.5. Survival Analysis According to the EASIX Score

**Figure 6:**
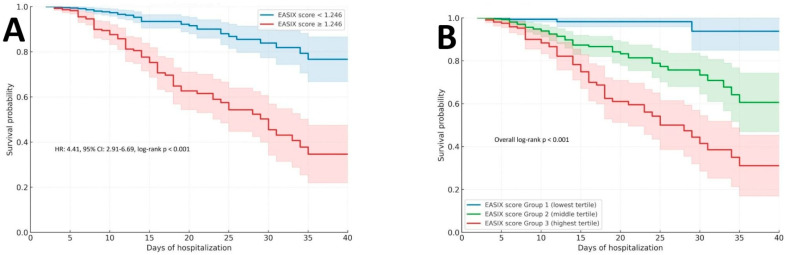
(**A**) Kaplan–Meier survival curves comparing patients with EASIX score <1.246 and ≥1.246. Shaded areas represent 95% confidence intervals. Mortality risk was significantly higher in the elevated EASIX group (HR 4.41, 95% CI 2.91–6.69; log-rank *p* < 0.001); (**B**) Kaplan–Meier survival curves across EASIX score tertiles defined as: Group 1 (lowest tertile, EASIX ≤ 0.677), Group 2 (middle tertile, 0.677 < EASIX < 1.466), and Group 3 (highest tertile, EASIX ≥ 1.466). Shaded bands indicate 95% confidence intervals. Survival differed significantly across tertiles (overall log-rank *p* < 0.001). Abbreviations: EASIX, endothelial activation and stress index.

As shown in Figure 6A, patients with an EASIX score ≥1.246 demonstrated significantly lower in-hospital survival probability compared to those with scores below this threshold. The survival curves diverged early during hospitalization and remained distinctly separated throughout follow-up. The HR for in-hospital mortality in the higher EASIX group was 4.41 (95% CI: 2.91–6.69; log-rank *p* < 0.001), indicating a significantly higher risk of in-hospital mortality with elevated EASIX values. Complementing this binary stratification, Figure 6B presents Kaplan–Meier curves for EASIX tertiles. A clear gradient of declining survival was observed from the lowest to the highest tertile groups, with a statistically significant difference across all groups (overall log-rank *p* < 0.001). Patients in the highest tertile (Group 3) experienced markedly worse outcomes compared to those in the lowest tertile, reinforcing the dose–response relationship between EASIX elevation and mortality risk.

## 4. Discussion

In this retrospective analysis of a real-world cohort of hospitalized patients with ADHF-rEF, we demonstrated that the EASIX, calculated upon admission, is independently associated with in-hospital mortality. Beyond its individual prognostic capacity, EASIX significantly improved risk classification when integrated into both clinical and laboratory-based multivariable models. Importantly, our findings revealed a clear dose–response relationship between rising EASIX values and in-hospital mortality, with the highest tertile associated with a mortality rate exceeding 25% and an over fourfold elevated hazard compared to the lowest tertile. To our knowledge, this is the first study to evaluate the prognostic performance of the EASIX score in the setting of ADHF-rEF, extending its clinical relevance from chronic cardiovascular contexts into the acute care domain. Consistent with its moderate ROC-based discrimination, EASIX should be interpreted as a complementary prognostic marker rather than a stand-alone diagnostic tool.

Serum creatinine has long been recognized as a powerful prognostic marker in ADHF-rEF, with its association with adverse outcomes consistently demonstrated across multiple prior studies [15]. Accordingly, its superior discriminative performance in ROC-based analyses within our cohort is not unexpected. The EASIX score, by contrast, represents a relatively novel composite index that integrates renal dysfunction with markers of cellular injury and hematologic stress. While its ROC-based discrimination was lower than that of creatinine alone, EASIX nonetheless showed meaningful discriminatory ability, exceeded that of LDH and platelet count, and remained independently associated with in-hospital mortality after adjustment for established clinical and laboratory predictors. These findings support the potential value of EASIX as a complementary, integrative marker reflecting systemic stress rather than a single-organ parameter.

Importantly, although non-survivors in our cohort exhibited clustering of established high-risk features—such as elevated NT-proBNP levels and a higher prevalence of CKD and DM [16]—EASIX remained independently associated with in-hospital mortality after adjustment for these factors. This suggests that EASIX does not merely recapitulate conventional markers of disease severity, but rather captures an overlapping yet distinct dimension of acute systemic vulnerability. Prior studies have similarly shown that composite indices of endotheliopathy retain prognostic value even after accounting for traditional cardiac and renal risk factors [17], supporting the concept that endothelial stress represents a unifying pathophysiological axis not fully reflected by organ-specific biomarkers alone [18].

The present findings underscore the prognostic importance of endothelial activation and injury in ADHF-rEF. Endothelial dysfunction has long been implicated in HF progression, contributing to impaired vasodilation, inflammatory activation, microvascular thrombosis, and consequent organ congestion [19]. The EASIX score provides a quantitative gauge of this pathophysiological stress by integrating three routine biomarkers originally formulated to assess endotheliopathy severity [20]. Each component may indirectly reflect a facet of endothelial perturbation: vascular injury and hemolysis release LDH into the circulation, renal hypoperfusion and microvascular compromise raise creatinine, and endothelial injury with complement activation leads to platelet consumption and thrombocytopenia [21]. In ADHF-rEF, a high EASIX could be proposed as a marker of global endothelial dysregulation, plausibly linked to profound hemodynamic compromise and the accompanying systemic inflammatory response [22]. This mechanistic link provides a plausible explanation for why patients with elevated EASIX in our cohort experienced markedly worse in-hospital outcomes. Notably, this concept is consistent with prior observations that global endotheliopathy indices, such as EASIX, correlate with adverse outcomes across diverse critical illnesses and cardiovascular conditions [23]. It should be emphasized that EASIX does not directly measure endothelial function, but rather represents a composite surrogate derived from routine laboratory parameters that are biologically linked to endothelial stress.

Our results align with and extend the emerging literature on EASIX in chronic and critical HF. In a recent chronic HF registry, Estler et al. showed that baseline EASIX was an independent predictor of 5-year mortality, even after adjustment for age, New York Heart Association class, NT-proBNP, and other conventional risk markers [10]. Our findings in ADHF-rEF complement this evidence from chronic HF by showing that even short-term, in-hospital outcomes are associated with the EASIX score at presentation. It is noteworthy that EASIX showed moderate correlation with NT-proBNP and clinical severity in the chronic HF study [10], suggesting that it captures a distinct aspect of disease biology. In our ADHF-rEF cohort, EASIX demonstrated numerically higher discriminative ability than NT-proBNP, CRP, and age for in-hospital mortality, and significantly improved risk reclassification when added to established clinical and laboratory models. This suggests that EASIX provides incremental prognostic information beyond conventional markers, potentially reflecting a unique pathophysiological domain related to acute endothelial dysfunction. Taken together, the chronic and acute data affirm that EASIX may provide prognostic information beyond traditional cardiac biomarkers, likely reflecting the burden of systemic microvascular dysfunction in HF patients.

Beyond HF per se, our findings resonate with studies of EASIX in other cardiovascular contexts, such as valvular heart disease and CAD. In patients undergoing transcatheter aortic valve replacement for severe aortic stenosis, EASIX has emerged as a meaningful risk marker of longer-term outcomes [24]. In a large retrospective CAD cohort, EASIX predicted mortality after coronary catheterization [11]. In aggregate, these observations across cardiovascular disease states support the broader premise that EASIX captures systemic microvascular and endothelial stress that may not be fully reflected by cardiac-specific biomarkers alone. In ADHF-rEF, where hemodynamic instability and systemic inflammation commonly coexist [25], this score may reflect a parallel biological vulnerability. In our consecutive ADHF-rEF cohort, patients with higher EASIX exhibited a steeper gradient in in-hospital mortality and earlier divergence in survival, suggesting that admission-based EASIX may similarly track acute systemic injury superimposed on HF decompensation. The markedly worse in-hospital outcomes observed in patients with elevated EASIX within our cohort may therefore be interpreted as the clinical correlate of this hypothesized endothelial–hemodynamic–inflammatory axis of stress at presentation. While causality cannot be confirmed, the concordance between EASIX and mortality across diverse cardiovascular settings lends biological and prognostic plausibility to its adverse associations in ADHF-rEF.

From a clinical standpoint, our study reinforces the potential utility of EASIX as an easily obtainable prognostic biomarker in ADHF-rEF. Its constituent variables are routinely measured upon hospital admission, making the score readily available without additional cost or specialized testing. In contrast to complex risk models that can be cumbersome at the bedside, EASIX offers a simple, objective measure of endothelial stress that could be incorporated into decision-making algorithms. In this context, the relatively high NPV and modest PPV observed for the EASIX cut-off merit consideration. These findings suggest that EASIX may be particularly useful for identifying patients at lower short-term risk, while elevated values may help flag a subgroup warranting closer clinical surveillance. Rather than serving as a stand-alone trigger for aggressive intervention, EASIX may assist in early risk stratification by informing the intensity of monitoring and allocation of in-hospital resources. Importantly, the main strength of EASIX in our ADHF-rEF cohort became evident when it was integrated with established prognostic axes rather than interpreted in isolation. The substantial NRI gains observed upon its addition to both clinical and laboratory-based models suggest that EASIX may best serve as a complementary systemic stress biomarker, refining early risk classification, informing clinical vigilance, and potentially contextualizing the severity of extracardiac vulnerability layered on impaired left ventricle performance.

## 5. Study Limitations

The primary limitations of this study relate to its single-center, retrospective observational design, which inherently restricts causal interpretation and leaves room for unmeasured confounding despite consecutive patient inclusion and comprehensive statistical adjustment. Our cohort exclusively comprised acutely decompensated HF patients with reduced LVEF admissions, without representation of mildly reduced or preserved ejection fraction acute HF, limiting generalizability across the full spectrum of HF phenotypes and impeding extrapolation to non-reduced LVEF presentations. In addition, we did not perform etiology-specific subgroup analyses (e.g., ischemic versus non-ischemic or valvular heart failure), and therefore cannot determine whether the short-term prognostic performance of EASIX varies according to the underlying cause of left ventricular dysfunction. EASIX was calculated only at hospital admission, and we did not perform temporal or longitudinal reassessment of the score, which prevents evaluation of dynamic changes in endothelial stress during the inpatient course and may underestimate its complexity as a biologically evolving signal. The study population demonstrated a pronounced male predominance, which creates additional constraints on external applicability given known sex-related differences in HF biology, systemic congestion, and inflammatory activation; consequently, sex-stratified prognostic analyses were not feasible due to limited female sample size and the number of outcome events. In addition, several clinically important in-hospital severity indicators—such as the need for invasive ventilation, inotropic support, or mechanical circulatory assistance [26,27]—were not consistently available in the dataset and therefore could not be incorporated into the analyses. These parameters are recognized as powerful short-term prognostic markers in ADHF-rEF [26,27] and may have provided further granularity if available. Likewise, established ADHF clinical phenotypes such as the wet–warm/dry–cold classification could not be reliably assessed due to inconsistent documentation of congestion and perfusion findings in this retrospective cohort. Furthermore, clinical severity indices beyond routine laboratory markers—such as lactate, interleukin profiles, or markers of venous congestion—were not consistently available for analysis, precluding deeper characterization of the acute systemic stress state potentially approximated by EASIX. Long-term outcomes and post-discharge cardiac functional status were beyond the scope of the present admission-based analysis; therefore, no conclusions regarding the prognostic implications of EASIX beyond the in-hospital period can be drawn from this study. We reported continuous NRI because no universally accepted, clinically validated in-hospital mortality risk categories exist for ADHF-rEF, and category-based NRI is highly dependent on arbitrarily chosen cut-points. Continuous NRI provides a threshold-free assessment of whether adding EASIX increases the predicted risk among patients who die and decreases it among survivors, reflecting improved risk ordering. However, continuous NRI may overstate incremental value and does not directly inform clinically actionable risk thresholds or calibration; therefore, it should be interpreted alongside discrimination metrics (e.g., AUC), and future studies should evaluate clinical utility using calibration and decision-analytic approaches. Finally, validation of these findings in prospective and multicenter ADHF-rEF cohorts remains necessary to confirm whether the additive prognostic performance observed for EASIX in our HF population extends to broader health-care settings and mixed-phenotype acute HF populations.

## 6. Conclusions

In this consecutive cohort of patients hospitalized for ADHF-rEF, the elevated EASIX score at admission was independently associated with a higher risk of in-hospital mortality. Higher admission-based EASIX values significantly improved mortality risk classification when combined with established clinical and laboratory predictors. External validation in prospective, multicenter ADHF-rEF cohorts encompassing diverse HF phenotypes is needed to confirm the generalizability and clinical applicability of these findings.

## Figures and Tables

**Table 1:** Baseline clinical, laboratory, and demographic characteristics of survivors and non-survivors.

Variables	Survivors (*n* = 745)	Non-Survivors (*n* = 105)	*p*-Value
Demographics and vital signs			
*Age*, (*years*)	63.7 ± 13.2	71.8 ± 11.2	<0.001
*Male gender*, *n* (%)	530 (71.1)	78 (74.3)	0.504
*Smoking*, *n* (%)	288 (38.7)	36 (34.3)	0.388
*Systolic blood pressure*, (mmHg)	124 ± 14	126 ± 14	0.312
*Heart rate*, (bpm)	80 ± 12	81 ± 12	0.358
Comorbidities, *n* (%)			
*Diabetes mellitus*	265 (35.6)	56 (53.3)	<0.001
*Hypertension*	462 (62.0)	71 (67.6)	0.266
*Coronary artery disease*	331 (44.4)	65 (61.9)	0.001
*Atrial fibrillation*	135 (18.1)	22 (21.0)	0.482
*Chronic kidney disease*	224 (30.1)	48 (45.7)	0.001
*Peripheral artery disease*	119 (16.0)	19 (18.1)	0.581
*COPD*	94 (12.6)	15 (14.3)	0.632
*Cardiac device* (*CRT or ICD*)	177 (23.8)	25 (23.8)	0.991
Baseline medications at presentation, *n* (%)			
*β-blocker*	504 (67.7)	78 (74.3)	0.171
*ACE-i or ARB*	410 (55.0)	65 (61.9)	0.184
*MRA*	301 (40.4)	39 (37.1)	0.523
*Antiaggregant*	294 (39.5)	37 (35.2)	0.406
*Anticoagulant*	151 (20.3)	19 (18.1)	0.602
*Statin*	203 (27.2)	29 (27.6)	0.936
Laboratory findings			
*LVEF*, (%)	29.2 ± 8.2	30.0 ± 8.3	0.362
*NT-proBNP*, (pg/mL)	1070 (121–35,000)	2016 (136–35,000)	<0.001
*Blood glucose*, (mg/dL)	114 (96–153)	138 (105–181)	0.001
*Serum creatinine*, (mg/dL)	1.1 (0.3–6.8)	1.6 (0.5–8.0)	<0.001
*Serum albumin*, (g/dL)	3.7 ± 0.6	3.7 ± 0.7	0.480
*Serum sodium*, (mmol/L)	138.0 ± 4.3	136.7 ± 4.3	0.004
*Serum potassium*, (mmol/L)	4.4 ± 0.6	4.3 ± 0.6	0.427
*Serum calcium*, (mg/dL)	9.0 ± 0.7	9.0 ± 0.9	0.886
*Serum chloride*, (mmol/L)	100.0 ± 7.5	99.0 ± 11.0	0.188
*LDH*, (U/L)	207.1 ± 40.4	221.6 ± 25.1	<0.001
*Hemoglobin*, (g/dL)	13.1 ± 2.4	11.8 ± 1.6	<0.001
*White blood cell*, (×10^9^/L)	8.4 (1.9–22.8)	8.8 (2.5–24.0)	0.174
*Neutrophil*, (×10^9^/L)	5.4 (0.9–15.1)	5.5 (1.8–17.7)	0.756
*Lymphocyte*, (×10^9^/L)	1.6 (0.2–4.9)	1.4 (0.1–4.1)	0.246
*Platelet*, (×10^9^/L)	239 (80–472)	225 (72–444)	0.080
*C-reactive protein*, (mg/L)	15 (5–34)	23 (4–47)	<0.001
*EASIX score*	0.94 (0.18–2.38)	1.49 (0.49–3.61)	<0.001
**Length of hospitalization, (days)**	9 (2–51)	13 (3–35)	0.001

Abbreviations: ACE-i, angiotensin-converting enzyme inhibitor; ARB, angiotensin-II receptor blocker; bpm, beats per minute; COPD, chronic obstructive pulmonary disease; CRT, cardiac resynchronization therapy; EASIX, endothelial activation and stress index; ICD, implantable cardioverter defibrillator; MRA, mineralocorticoid receptor antagonist; LDH, lactate dehydrogenase; LVEF, left ventricular ejection fraction; NT-proBNP, N-terminal pro B-type natriuretic peptide.

**Table 2:** Univariable and multivariable logistic regression analyses showing predictors of in-hospital mortality in patients with acute decompensated heart failure and reduced ejection fraction.

Variables	Univariable Regression		Multivariable Regression	
	Odds Ratio (95% Confidence Interval)	*p*-Value	Odds Ratio (95% Confidence Interval)	*p*-Value
Age	1.055 (1.035–1.074)	<0.001	1.045 (1.025–1.065)	<0.001
Diabetes mellitus	2.070 (1.371–3.125)	<0.001	2.115 (1.336–3.349)	0.001
Coronary artery disease	2.032 (1.336–3.092)	<0.001	1.512 (0.951–2.406)	0.081
Chronic kidney disease	1.959 (1.294–2.965)	0.001	1.652 (1.039–2.628)	0.034
NT-proBNP, (per 500 pg/mL)	1.029 (1.015–1.043)	<0.001	1.020 (1.003–1.036)	0.021
Blood glucose	1.675 (1.118–2.542)	0.002	–	–
Serum creatinine	2.398 (1.832–3.138)	<0.001	–	–
Serum sodium	0.933 (0.890–0.978)	0.004	0.945 (0.899–0.993)	0.025
LDH	1.010 (1.004–1.015)	<0.001	–	–
Hemoglobin, (per 1 g/dL)	0.774 (0.703–0.851)	<0.001	0.832 (0.749–0.924)	<0.001
Platelet	0.998 (0.995–1.000)	0.109	–	–
C-reactive protein	1.005 (1.002–1.007)	<0.001	1.005 (1.002–1.008)	<0.001
EASIX score	1.339 (1.184–1.514)	<0.001	1.273 (1.123–1.442)	<0.001

Abbreviations: EASIX, endothelial activation and stress index; LDH, lactate dehydrogenase; NT-proBNP, N-terminal pro B-type natriuretic peptide.

**Table 3:** Clinical and laboratory characteristics across EASIX score tertiles.

Variables	EASIX Score Group 1 (Lowest Tertile) (*n* = 212)	EASIX Score Group 2 (Middle Tertile) (*n* = 424)	EASIX Score Group 3 (Highest Tertile) (*n* = 214)	*p*-Value
Demographics and vital signs				
*Age*, (*years*)	60.6 ± 13.4 ^a^	64.6 ± 13.0 ^b^	68.9 ± 12.0 ^c^	<0.001
*Male gender*, *n* (%)	127 (59.9) ^a^	327 (77.1) ^b^	154 (72.0) ^b^	<0.001
*Smoking*, *n* (%)	77 (36.3)	170 (40.1)	77 (36.0)	0.495
*Systolic blood pressure*, (mmHg)	126 ± 16	125 ± 15	124 ± 13	0.549
*Heart rate*, (bpm)	80 ± 12	80 ± 12	80 ± 11	0.888
Comorbidities, *n* (%)				
*Diabetes mellitus*	76 (35.8)	162 (38.2)	83 (38.8)	0.794
*Hypertension*	120 (56.6)	272 (64.2)	141 (65.9)	0.096
*Coronary artery disease*	87 (41.0) ^a^	189 (44.6) ^a^	120 (56.1) ^b^	0.004
*Atrial fibrillation*	29 (13.7)	87 (20.5)	41 (19.2)	0.321
*Chronic kidney disease*	63 (29.7) ^a^	122 (28.8) ^a^	87 (40.7) ^b^	0.007
*Peripheral artery disease*	32 (15.1)	72 (17.0)	34 (15.9)	0.821
*COPD*	14 (6.6) ^a^	52 (12.3) ^b^	43 (20.1) ^c^	<0.001
*Cardiac device* (*CRT or ICD*)	50 (23.6)	94 (22.2)	58 (27.1)	0.384
Baseline medications at presentation, *n* (%)				
*β-blocker*	131 (61.8) ^a^	287 (67.7) ^a^	164 (76.6) ^b^	0.004
*ACE-i or ARB*	110 (51.9)	242 (57.1)	123 (57.5)	0.399
*MRA*	90 (42.5)	166 (39.2)	84 (39.3)	0.702
*Antiaggregant*	81 (38.2)	167 (39.4)	83 (38.8)	0.958
*Anticoagulant*	29 (13.7) ^a^	84 (19.8) ^a^	57 (26.6) ^b^	0.004
*Statin*	47 (22.2)	127 (30.0)	58 (27.1)	0.115
Laboratory findings				
*LVEF*, (%)	29.2 ± 8.4	29.4 ± 8.4	29.2 ± 7.8	0.965
*NT-proBNP*, (pg/mL)	968 (122–35,000) ^a^	1164 (121–35,000) ^b^	1511 (127–35,000) ^c^	<0.001
*Blood glucose*, (mg/dL)	111 (94–144) ^a^	117 (99–160) ^b^	120 (96–161) ^b^	0.042
*Serum albumin*, (g/dL)	3.8 ± 0.6 ^a^	3.8 ± 0.6 ^a^	3.6 ± 0.7 ^b^	<0.001
*Serum sodium*, (mmol/L)	138.6 ± 3.7 ^a^	137.7 ± 4.0 ^a,b^	137.3 ± 5.2 ^b^	0.009
*Serum potassium*, (mmol/L)	4.4 ± 0.5	4.4 ± 0.6	4.4 ± 0.7	0.568
*Hemoglobin*, (g/dL)	13.3 ± 2.1 ^a^	13.1 ± 2.3 ^a^	12.1 ± 2.2 ^b^	<0.001
*White blood cell*, (×10^9^/L)	8.0 (1.8–22.7)	8.2 (2.4–24)	8.2 (3.0–20.3)	0.501
*Neutrophil*, (×10^9^/L)	5.3 (1.7–11.2)	5.3 (1.1–15.1)	5.8 (0.9–15.1)	0.247
*Lymphocyte*, (×10^9^/L)	2.0 (0.4–4.4) ^a^	2.0 (0.01–4.8) ^a^	1.4 (0.2–4.9) ^b^	<0.001
*C-reactive protein*, (mg/L)	14 (5–34) ^a^	26 (4–33) ^b^	30 (5–37) ^b^	0.011
**In-hospital mortality, *n* (%)**	3 (1.4) ^a^	46 (10.8) ^b^	56 (26.2) ^c^	<0.001
**Length of hospitalization, (days)**	9 (3–47)	9 (2–51)	10 (3–51)	0.936

Abbreviations: ACE-i, angiotensin-converting enzyme inhibitor; ARB, angiotensin-II receptor blocker; bpm, beats per minute; COPD, chronic obstructive pulmonary disease; CRT, cardiac resynchronization therapy; EASIX, endothelial activation and stress index; ICD, implantable cardioverter defibrillator; MRA, mineralocorticoid receptor antagonist; LVEF, left ventricular ejection fraction; NT-proBNP, N-terminal pro B-type natriuretic peptide. Different superscripts indicate the statistical difference between groups. Superscript letters (a, b, c) indicate the results of pairwise post hoc comparisons between OPS groups. Groups that share the same letter are not significantly different from each other, whereas groups with different letters show statistically significant differences (*p* < 0.05).

## Data Availability

Data are available from the corresponding author upon reasonable request. The data are not publicly available due to privacy or ethical restrictions.
